# Prevalence and associated risk factors for hypovitaminosis D in patients scheduled for primary total hip arthroplasty in Germany

**DOI:** 10.1177/11207000261437108

**Published:** 2026-05-06

**Authors:** Jan Oberfeld, Lisa Wiederhold, Miledi Hoxha, Tizian Heinz, Maximilian Rudert, Felix Hochberger, Manuel Weissenberger, Konstantin Horas

**Affiliations:** Orthopedic Clinic König-Ludwig-Haus, Würzburg, Germany

**Keywords:** BMI, hypovitaminosis D, risk factors, total hip arthroplasty, prehabilitation, supplementation, vitamin D

## Abstract

**Background:**

Hypovitaminosis D is widespread in orthopaedic patients and may influence outcomes following joint arthroplasty. However, data specifically addressing vitamin D status in patients undergoing primary total hip arthroplasty (THA) remain limited. This study aimed to determine the prevalence of hypovitaminosis D and identify associated risk factors in a large German THA cohort.

**Methods:**

In this prospective single-centre study, 679 consecutive patients scheduled for primary THA due to osteoarthritis were evaluated over a 12-month period. Preoperative serum 25-hydroxyvitamin D (25[OH]D), parathyroid hormone, and calcium levels were measured. Vitamin D status was categorised as deficiency (⩽19 ng/mL), insufficiency (20–29 ng/mL), or sufficiency (⩾30 ng/mL). Associations between vitamin D status and demographic, clinical, and seasonal variables were analysed using χ^2^ tests, ANOVA, and multivariable linear and logistic regression models.

**Results:**

The mean serum 25(OH)D level was 26.0 ± 13.2 ng/mL, and hypovitaminosis D was present in 66,3% of patients (33.9% deficiency, 32.4% insufficiency). Season of sampling showed no significant influence on vitamin D levels. In multivariable analysis, preoperative vitamin D supplementation was the strongest positive predictor of higher 25(OH)D levels (B = +16.1 ng/mL; *p* < 0.001), while higher BMI category (B = −1.64 ng/mL per category; *p* < 0.001) and male sex (B = −2.45 ng/mL; *p* = 0.010) were independently associated with lower levels. Logistic regression confirmed reduced odds of vitamin D sufficiency among men (OR 0.59; *p* = 0.007) and with increasing BMI category (OR 0.74; *p* = 0.002). Vitamin D status was not associated with smoking, comorbidity burden, season, or functional scores (WOMAC, EuroQol).

**Conclusions:**

Hypovitaminosis D is highly prevalent in German patients undergoing primary THA, independent of season. Obesity and male sex are significant risk factors, whereas supplementation is strongly protective. Routine preoperative assessment and targeted optimisation of vitamin D levels, particularly in high-risk subgroups, may represent a simple, cost-effective component of arthroplasty prehabilitation. Further research is needed to determine whether correcting vitamin D deficiency translates to improved postoperative outcomes.

## Introduction

In recent years, there has been a growing interest in investigating vitamin D serum levels among patients undergoing primary elective joint replacement surgery.^1,2^ This trend reflects an expanding understanding of vitamin D’s diverse physiological roles and its significance beyond bone health. While vitamin D is best known for regulating calcium homeostasis and bone mineralisation, it also contributes to muscle function and modulates various autoimmune and metabolic processes, influencing conditions such as diabetes, hypertension, multiple sclerosis and cancer.^3–8^ Vitamin D can be obtained through dietary intake (5–10%) and, more importantly, via cutaneous synthesis triggered by sunlight exposure (80–90%).^9,10^ Ultraviolet B (UVB) photons convert 7-dehydrocholesterol in the skin to precholecalciferol, which is subsequently transformed into vitamin D3 (cholecalciferol).^11^ In the liver, vitamin D3 is then hydroxylated to 25-hydroxyvitamin D (25[OH]D), the primary circulating form of vitamin D. A further hydroxylation step in the kidney, catalysed by the enzyme 1α-hydroxylase, produces calcitriol (1,25[OH]2D), the biologically active form.^12^ Calcitriol acts via specific vitamin D receptors to regulate calcium metabolism and influence cell differentiation and proliferation.^6^ Globally, vitamin D deficiency (hypovitaminosis D) is recognised as the most common nutritional deficiency, affecting over 1 billion people worldwide.^6,11,13^ At the same time, the number of patients requiring primary joint replacement due to advanced osteoarthritis is expected to rise sharply.^14^ Given the high prevalence of hypovitaminosis D, these projections suggest that a considerable proportion of patients undergoing primary total hip arthroplasty are likely to have suboptimal vitamin D levels. Moreover, some evidence indicates a potential relationship between vitamin D deficiency and the progression of osteoarthritis.^15^ The prevalence of hypovitaminosis D varies considerably depending on geographic location, season, and dietary vitamin D fortification.^11,16^ Although several studies have examined vitamin D status in general orthopaedic populations, or lower extremity total joint arthroplasty, data specifically focused on patients undergoing primary THA remain limited.^17–19^ Importantly, low serum vitamin D levels have been associated with increased hospitalisation rates and poorer postoperative outcomes in orthopaedic surgery.^20–22^ Therefore, the primary aim of this study was to determine the prevalence of hypovitaminosis D and to identify its associated risk factors in a large, single-centre cohort of patients scheduled for primary total hip arthroplasty.

## Materials and methods

### Study design and setting

This prospective study was approved by the local ethics committee (approval number: 1/23-me). Written informed consent was obtained from all participants prior to inclusion. Patients were prospectively evaluated and recruited from a single orthopedic university centre located at a latitude of 49.5° north. Enrollment occurred continuously throughout the year to account for potential seasonal variations in vitamin D levels.

### Patient cohort

All patients scheduled for primary total hip arthroplasty (THA) due to osteoarthritis of the hip were eligible for inclusion. The final study cohort consisted of 679 patients in total. Recruitment commenced on the 17 May 2023 and concluded 1 year later. Patients undergoing surgery for acute fractures or presenting with contraindications to vitamin D supplementation were excluded. In addition, patients who declined to provide informed consent were not included in the analysis. In cases where vitamin D deficiency was detected, supplementation began on the first postoperative day, consisting of 20,000 IU twice, followed by 1000 IU daily thereafter. Demographic variables (age, sex, body mass index [BMI], comorbidities, renal function, medication) and administrative parameters (length of hospital stay, readmissions) were collected for all participants. Preoperative joint-specific function and health-related quality of life were assessed using Western Ontario and McMaster Universities Osteoarthritis Index (WOMAC) and EuroQol scores (EQ-5D VAS scale [0–100]).

### Vitamin D serum level measurements

Serum concentrations of 25-hydroxyvitamin D (25[OH]D), parathyroid hormone (PTH), and calcium were measured for all patients as part of the routine preoperative blood work, typically performed the day before surgery. Eligibility screening was carried out during preoperative admission consultations by attending physicians. Blood samples were obtained throughout all 4 seasons to capture potential seasonal variations. Measurements were conducted in the in-house laboratory using the cobas 25-Hydroxyvitamin D Assay (vitamin D total) and the Elecsys PTH (1–84) Assay on the cobas e411 Analyzer (Roche Diagnostics, Mannheim, Germany).

Serum 25(OH)D levels were further classified according to the US Endocrine Society of Medicine:^23^ (1) Deficiency: ⩽19 ng/mL; (2) Insufficiency: 20–29 ng/mL; and (3) Sufficiency: ⩾30 ng/mL.

### Surgical procedure

All procedures were performed using the minimally-invasive anterior approach to the hip. For uncemented fixation, an ML Taper stem (Zimmer Biomet, Warsaw, IN, USA) was implanted, whereas cemented cases received an original Müller-Geradschaft stem (Zimmer Biomet). The acetabular component used in all cases was the Allofit S-Alloclassic Acetabular System cup (Zimmer Biomet). Where cemented fixation was required, stems were implanted using Palacos R+G bone cement (Heraeus Medical, Wehrheim, Germany). Postoperatively, full weight-bearing as tolerated was permitted, and early functional rehabilitation was initiated. A total of 1.5 g of tranexamic acid was administered intraoperatively, followed by 500 mg intravenously 5–6 hours postoperatively, in line with the institutional fast-track protocol. Surgical drains were generally avoided unless clinically indicated.

### Statistical analysis

Statistical analyses were performed using IBM SPSS Statistics for Windows, Version 30.0.0.0 (IBM Corp., Armonk, NY, USA, 2024). Categorical variables were summarized as absolute and relative frequencies, whereas continuous variables were expressed as mean ± standard deviation (SD) and, where appropriate, median and interquartile range. Normality of distribution was assessed using Kolmogorov–Smirnov and Shapiro-Wilk tests. Depending on the distribution, group comparisons were conducted with independent-samples *t*-tests or 1-way ANOVA for approximately normally distributed data, and Mann-Whitney U-tests for non-normally distributed data. Differences in categorical variables (e.g. vitamin D status across seasons or BMI categories) were evaluated using the Chi-square test, where appropriate, effect size measures (e.g. Cohen’s d) were calculated to aid interpretation.

To identify independent predictors of preoperative 25(OH)D serum levels, a multiple linear regression model was constructed including age, BMI category, sex, season of sampling, smoking status, comorbidity groups, medication classes and regular vitamin D supplementation as explanatory variables (categorical predictors entered as dummy variables). Model assumptions (linearity, homoscedasticity, normality of residuals and absence of multicollinearity) were evaluated using residual plots, variance inflation factors, the Durbin-Watson statistic and Cook’s distance. In a second step, a binary logistic regression analysis was performed with hypovitaminosis D (deficiency/insufficiency) versus sufficiency as the dependent variable, using the same set of predictors to estimate adjusted odds ratios (ORs) and 95% confidence intervals (CIs). Model adequacy for the logistic regression was assessed using the Hosmer-Lemeshow goodness-of-fit test, pseudo-R^2^ statistics (Cox & Snell, Nagelkerke) and overall classification accuracy. Group differences between patients with hypovitaminosis D and those with sufficient vitamin D levels were analyzed using Mann-Whitney U-tests, and correlations between 25(OH)D and WOMAC/EuroQol were explored with Spearman rank correlation coefficients. All tests were 2-sided, a *p*-value < 0.05 was considered statistically significant, and analyses were based on complete cases; no imputation of missing data was undertaken.

## Results

### Patient characteristics

A total of 679 patients scheduled for primary total hip arthroplasty were included in the analysis. The mean age was 67.7 ± 10.7 years (median 68; range 22–96 years), and 370 patients (54.5%) were female. The mean BMI was 28.1 ± 5.3 kg/m^2^ (median 27.0; *n* = 653), with the majority of patients being overweight or obese (BMI ⩾ 25 kg/m^2^). Female patients had a mean BMI of 27.9 ± 5.6 kg/m^2^ and male patients 28.3 ± 4.9 kg/m^2^; BMI did not differ significantly between sexes (*p* > 0.05).

Active smoking was reported by 82 patients (12.1%), whereas 577 (85.0%) were non-smokers. Most patients did not present relevant chronic comorbidities; diabetes mellitus was present in 13.0%, rheumatoid disease in 2.1%, tumour disease in 2.4% and chronic obstructive pulmonary disease (COPD) in 1.6%; 0.9% had multiple of these conditions. Regarding renal function, 38.4% had no chronic kidney disease, 51.1% had CKD stage II (eGFR 60–89 mL/min/1.73 m^2^), and 10.5% CKD stage III–IV.

At baseline, 125 patients (18.4%) were already receiving vitamin D medication. Most procedures were performed with uncemented fixation (73.2%), while 26.8% received a hybrid construct. The mean length of hospital stay (LOS) was 4.5 ± 2.5 days (median 4; IQR 3–5; *n* = 676).

Preoperative joint-specific function and health-related quality of life were moderately impaired, with a mean WOMAC score of 55.4 ± 18.6 points and a mean EuroQol score of 12.4 ± 1.3 (both n ≈ 450). Both scores were slightly higher in women than in men (WOMAC 58.6 ± 19.2 vs. 51.7 ± 17.3; *p* < 0.001; EuroQol 12.66 ± 1.39 vs. 12.15 ± 1.13; *p* < 0.001), corresponding to small-to-moderate effect sizes (WOMAC Cohen’s d = 0.37; EuroQol Cohen’s d = 0.39). The baseline characteristics of the study cohort are summarised in Table 1.

**Table 1.** Baseline characteristics of the study cohort (*n* = 679).

Variable	Value
**Demographics**	
Age, years	67.7 ± 10.7 (median 68; range 22–96)
Sex, female	370 (54.5%)
Sex, male	309 (45.5%)
**Body mass index**	
BMI, kg/m^2^ (n = 653)	28.1 ± 5.3 (median 27.0; range 15.6–49.9)
BMI < 25 kg/m^2^	189/653 (28.9%)
BMI 25–29.9 kg/m^2^	273/653 (41.8%)
BMI 30–34.9 kg/m^2^	120/653 (18.4%)
BMI 35–39.9 kg/m^2^	51/653 (7.8%)
BMI ⩾ 40 kg/m^2^	20/653 (3.1%)
**Lifestyle and comorbidities**	
Current smoker	82 (12.1%)
Non-smoker	577 (85.0%)
No recorded chronic disease	544 (80.1%)
Diabetes mellitus	88 (13.0%)
Rheumatoid disease	14 (2.1%)
Tumour disease	16 (2.4%)
COPD	11 (1.6%)
Multiple comorbidities	6 (0.9%)
**Renal function (CKD category)**	
No CKD	261 (38.4%)
CKD stage II (eGFR 60–89)	347 (51.1%)
CKD stage III (eGFR 30–59)	67 (9.9%)
CKD stage IV (eGFR <30)	4 (0.6%)
**Medication**	
No medication	394 (58.0%)
Aromatase inhibitors	4 (0.6%)
Systemic glucocorticoids	28 (4.1%)
L-thyroxine	99 (14.6%)
Proton pump inhibitors	99 (14.6%)
Mixed medication regimen	55 (8.1%)
Pre-existing vitamin D supplementation	125 (18.4%)
**Surgical and hospital parameters**	
Uncemented fixation	497 (73.2%)
Hybrid fixation	182 (26.8%)
Length of stay (LOS), days (n = 676)	4.5 ± 2.5 (median 4; IQR 3–5; range 2–47)

BMI, body mass index; CKD, chronic kidney disease.

### Vitamin D status in the study cohort

The mean preoperative serum 25(OH)D level in the total cohort was 26.0 ± 13.2 ng/mL (median 24.0 ng/mL; IQR 16–34; range 3–92 ng/mL). Figure 1 illustrates the distribution of the vitamin D levels in the patient cohort. The distribution of 25(OH)D values deviated significantly from normality (Kolmogorov-Smirnov *p* < 0.001; Shapiro-Wilk *p* < 0.001).

**Figure 1.**
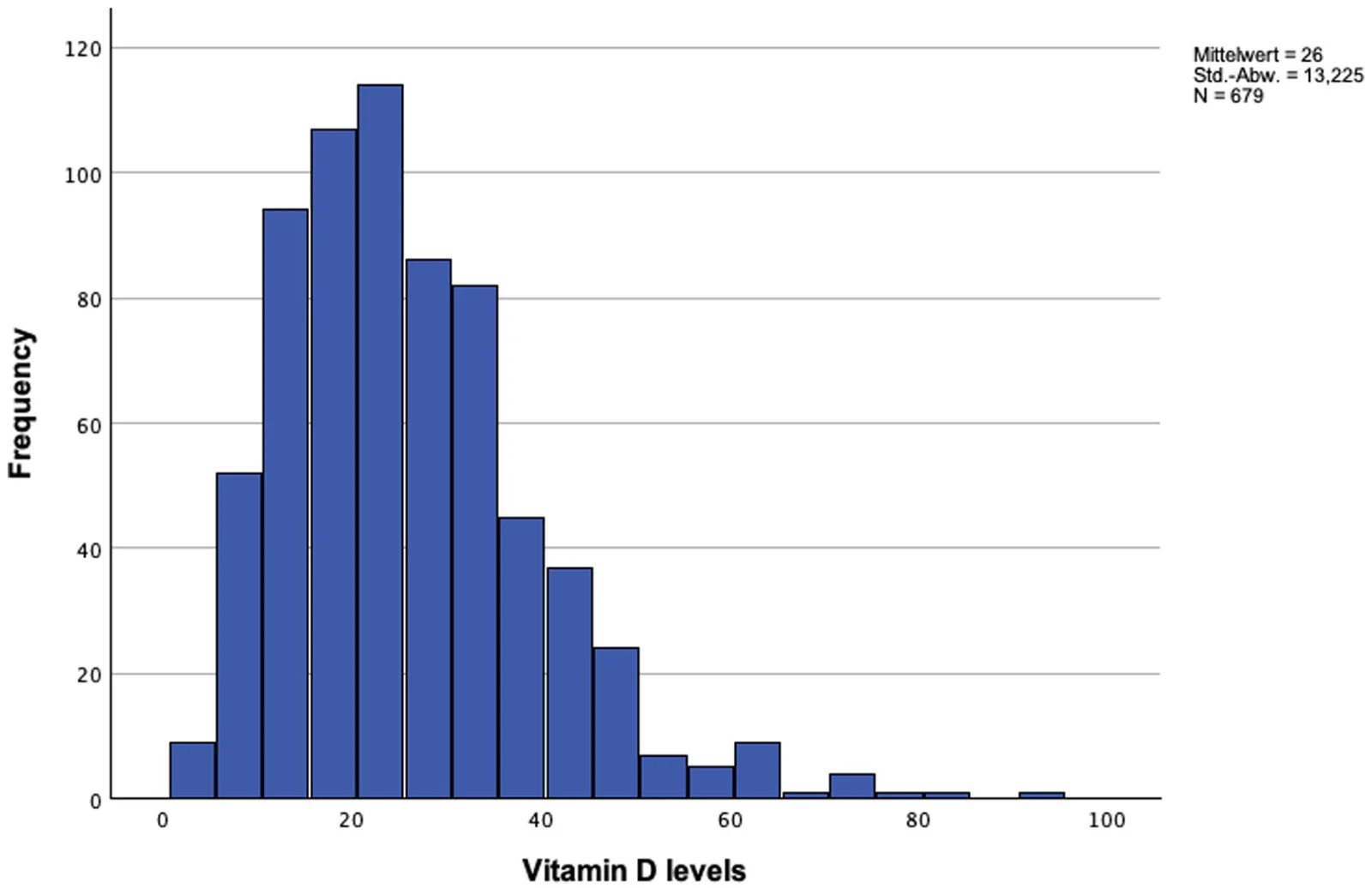
Histogram showing distribution of vitamin D levels.

According to the predefined cut-offs, 230 patients (33.9%) had vitamin D deficiency (⩽19 ng/mL), 220 (32.4%) had vitamin D insufficiency (20–29 ng/mL) and 229 (33.7%) had sufficient levels (⩾30 ng/mL). Overall, 462 of 679 patients (68.0%) presented with hypovitaminosis D (deficiency + insufficiency), which was significantly more frequent than vitamin D sufficiency (32.0%; χ^2^ test, *p* < 0.001). Blood sampling for 25(OH)D was distributed across all 4 seasons (spring 25.8%, summer 20.5%, autumn 27.1%, winter 26.7%).

### Associations between vitamin D status and clinical factors

In bivariate χ^2^ analyses, several demographic and clinical factors were evaluated for their association with hypovitaminosis D (vitamin D deficiency + insufficiency vs. sufficiency). The main results are summarised in Table 2.

**Table 2.** Prevalence of hypovitaminosis D according to selected clinical characteristics (*n* = 679; BMI *n* = 653, missing data in 26 cases).

Variable	Hypovitaminosis D *n* (%)	Vitamin D sufficiency *n* (%)	*p*-value (χ^2^, df)
**Sex**			χ^2^ = 22.58, df = 1, p < 0.001
Female (*n* = 370)	223 (60.3%)	147 (39.7%)	
Male (*n* = 309)	239 (77.3%)	70 (22.7%)	
**BMI category** (kg/m^2^)			χ^2^ = 15.45, df = 4, p = 0.004
<25 (*n* = 189)	110 (58.2%)	79 (41.8%)	
25–29.9 (*n* = 273)	193 (70.7%)	80 (29.3%)	
30–34.9 (*n* = 120)	89 (74.2%)	31 (25.8%)	
35–39.9 (*n* = 51)	41 (80.4%)	10 (19.6%)	
⩾40 (*n* = 20)	15 (75.0%)	5 (25.0%)	
**Preoperative vitamin D medication**			χ^2^ = 87.50, df = 1, p < 0.001
Yes (*n* = 125)	41 (32.8%)	84 (67.2%)	
No (*n* = 554)	421 (76.0%)	133 (24.0%)	
**CKD stage (eGFR, mL/min/1.73 m^2^)**			χ^2^ = 12.35, df = 3, p = 0.006
No CKD (*n* = 261)	190 (72.8%)	71 (27.2%)	
60–89 (*n* = 347)	227 (65.4%)	120 (34.6%)	
30–59 (*n* = 67)	45 (67.2%)	22 (32.8%)	
<29 (*n* = 4)	0 (0.0%)	4 (100.0%)	
**Season of sampling**			χ^2^ = 2.92, df = 3, p = 0.404
Spring (*n* = 175)	125 (71.4%)	50 (28.6%)	
Summer (*n* = 139)	99 (71.2%)	40 (28.8%)	
Autumn (*n* = 184)	120 (65.2%)	64 (34.8%)	
Winter (*n* = 181)	118 (65.2%)	63 (34.8%)	

BMI, body mass index; CKD, chronic kidney disease.

Vitamin D status was significantly associated with sex, BMI category, preoperative vitamin D supplementation and chronic kidney disease (CKD) stage. Women showed a higher proportion of vitamin D sufficiency compared to men (39.7% vs. 22.7%), while hypovitaminosis D was more frequent in male patients (77.3% vs. 60.3%; χ^2^ = 22.58, df = 1, *p* < 0.001). Across BMI categories, the prevalence of hypovitaminosis D increased significantly with higher BMI (Figure 2) (58.2% in BMI < 25 kg/m^2^, 70.7% in 25–29.9, 74.2% in 30–34.9, 80.4% in 35–39.9, 75.0% in ⩾ 40 kg/m^2^; χ^2^ = 15.45, df = 4, *p* = 0.004; linear trend χ^2^ = 12.14, df = 1, *p* < 0.001).

**Figure 2.**
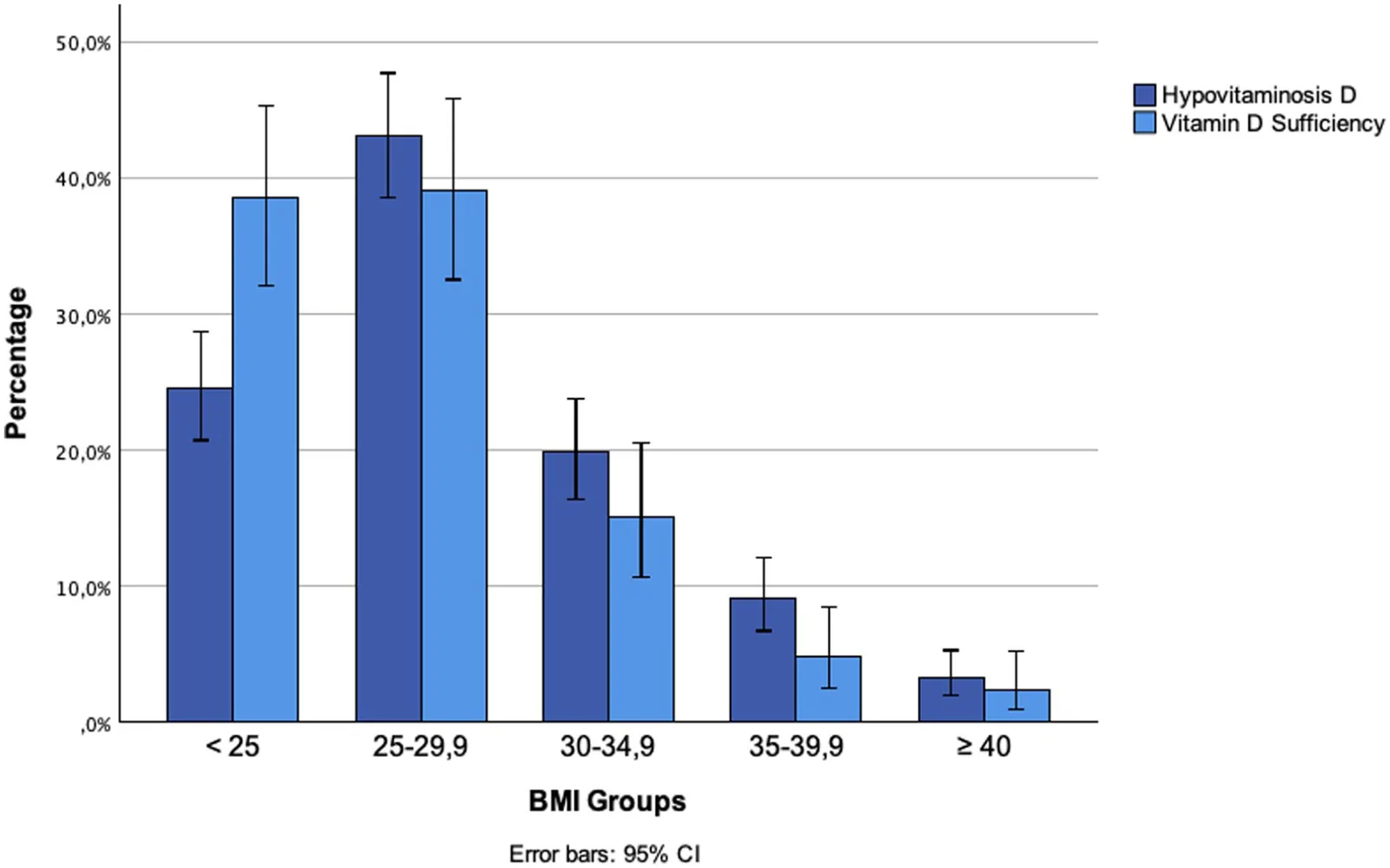
Vitamin D status in relation to BMI.

Preoperative vitamin D supplementation was strongly associated with a more favorable vitamin D status: among patients receiving vitamin D medication, 67.2% were sufficient and 32.8% had hypovitaminosis D, whereas in patients without supplementation 24.0% were sufficient and 76.0% had hypovitaminosis D (χ^2^ = 87.50, df = 1, *p* < 0.001). Chronic kidney disease stage was also related to vitamin D status (χ^2^ = 12.35, df = 3, *p* = 0.006), with a lower proportion of hypovitaminosis D in patients with severely reduced eGFR (<29 mL/min/1.73 m^2^) compared to those without or with only mildly reduced renal function.

In contrast, there was no significant association between hypovitaminosis D and season of blood sampling (spring 71.4%, summer 71.2%, autumn 65.2%, winter 65.2%; χ^2^ = 2.92, df = 3, *p* = 0.404) or smoking status (67.1% in smokers, 68.1% in non-smokers, χ^2^ = 0.07, df = 2, *p* = 0.965). Similarly, vitamin D status was not related to cementation technique (uncemented vs. hybrid; χ^2^ = 0.51, df = 1, *p* = 0.476), LOS (categorised in days; χ^2^ = 17.77, df = 15, *p* = 0.275), comorbidity category (χ^2^ = 8.43, df = 5, *p* = 0.134) or general medication class (χ^2^ = 7.29, df = 5, *p* = 0.200). When age was analysed in yearly categories, no significant association with vitamin D status was observed (χ^2^ = 57.66, df = 58, *p* = 0.488).

Complementary mean comparisons confirmed these patterns. In patients with available BMI data (*n* = 653), mean 25(OH)D levels decreased progressively with increasing BMI (from 28.3 ± 13.6 ng/mL in BMI <25 kg/m^2^ to 20.7 ± 12.6 ng/mL in BMI ⩾ 40 kg/m^2^; 1-way ANOVA F(4.648) = 4.30, *p* = 0.002; significant linear trend *p* < 0.001). In an exploratory dichotomization (BMI <25 vs. ⩾25 kg/m^2^), mean 25(OH)D did not differ significantly between groups (26.5 ± 12.4 vs. 25.9 ± 13.5 ng/mL; *p* = 0.30). Smoking status had no relevant impact on mean 25(OH)D (26.4 ± 14.4 ng/mL in smokers vs. 26.0 ± 13.1 ng/mL in non-smokers; *p* = 0.82).

Preoperative vitamin D supplementation showed the largest effect on serum 25(OH)D. Supplemented patients had markedly higher levels than non-supplemented patients (Figure 3) (39.8 ± 15.9 vs. 22.9 ± 10.2 ng/mL; *p* < 0.001, Cohen’s d ≈ 1.47). Consistently, the prevalence of hypovitaminosis D (deficiency or insufficiency) was substantially lower in the supplementation group (32.8%) compared to patients without supplementation (Figure 4) (76.0%; χ^2^ = 87.5, df = 1, *p* < 0.001).

**Figure 3.**
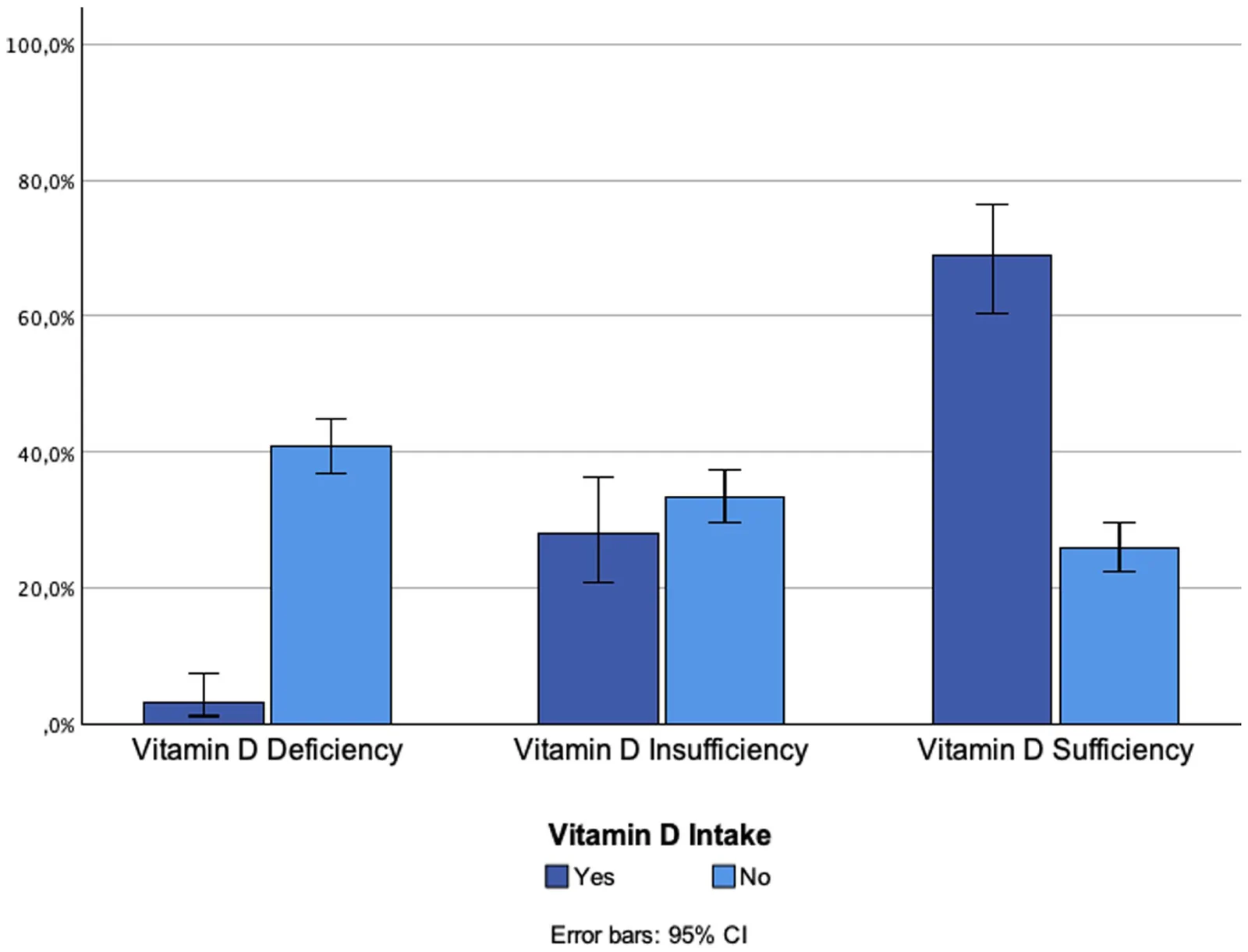
Prevalence of hypovitaminosis.

**Figure 4.**
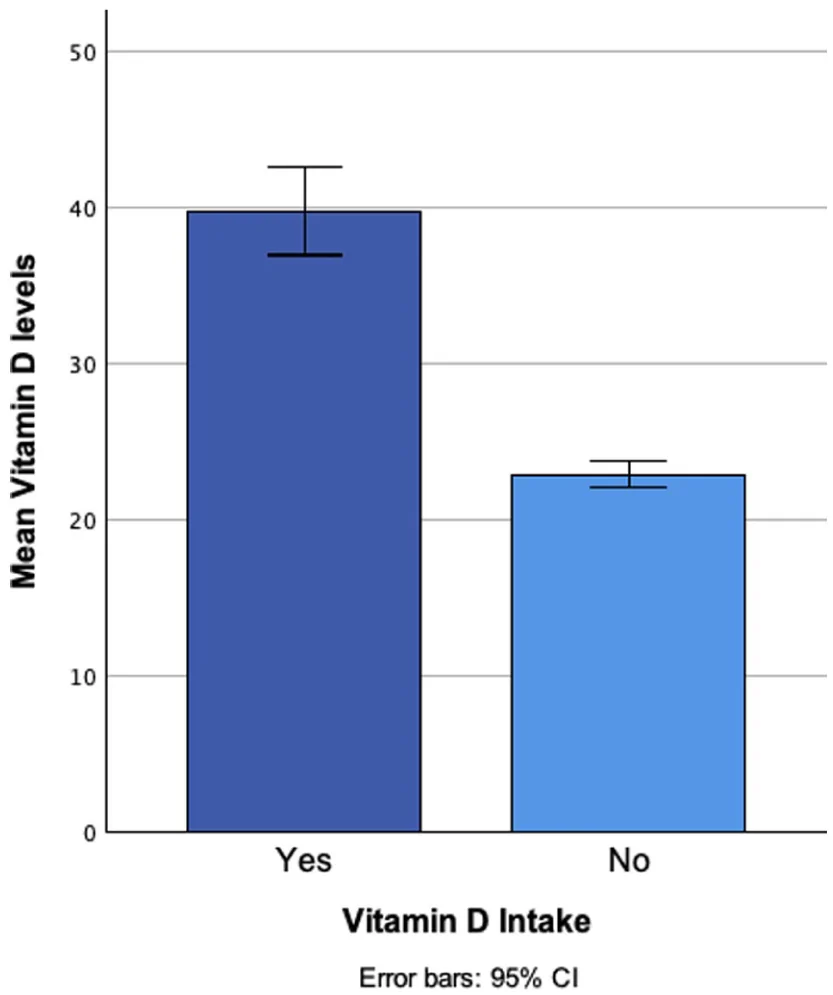
Mean vitamin D level in dependence of vitamin D intake.

Length of hospital stay (LOS) did not differ significantly between patients with hypovitaminosis D and those with sufficient 25(OH)D (median 4 days in both groups; 4.43 ± 1.90 vs. 4.71 ± 3.40 days; Mann-Whitney U-test = 46,295.5, *p* = 0.17). When stratified by vitamin D supplementation, patients with preoperative supplementation had a slightly longer LOS than those without (median 5 vs. 4 days; 5.20 ± 4.32 vs. 4.36 ± 1.81 days; Mann-Whitney U-test = 26,890.5, *p* < 0.001). Supplemented patients were also significantly older (72.2 ± 9.1 vs. 66.7 ± 10.8 years; *p* < 0.001), suggesting that vitamin D intake was more common among older patients.

Preoperative joint-specific function and health-related quality of life did not differ between patients with hypovitaminosis D and those with sufficient vitamin D levels. Median WOMAC and EuroQol scores were comparable in both groups (both *p* > 0.3, Mann-Whitney U-test), and there were no relevant correlations between 25(OH)D levels and WOMAC or EuroQol scores (Spearman |ρ| < 0.1).

### Multivariable analysis

#### Predictors of 25(OH)D serum levels

In a multiple linear regression model including age, sex, BMI categories, season of blood sampling, smoking status, comorbidities and preoperative vitamin D supplementation, the overall model explained about 30% of the variance in serum 25(OH)D (R^2^ = 0.30, adjusted R^2^ = 0.28; ANOVA: F(19,633) = 14.44, *p* < 0.001). There was no indication of relevant residual autocorrelation (Durbin-Watson = 1.98).

Preoperative vitamin D supplementation was by far the strongest positive predictor (B = 16.14 ng/mL; 95% CI, 13.76–18.52; *p* < 0.001), indicating that supplemented patients had, on average, 25(OH)D levels more than 16 ng/mL higher than comparable non-supplemented patients. Higher BMI categories were associated with lower vitamin D levels (B = −1.64 per BMI category; 95% CI, −2.52–−0.75; *p* < 0.001), i.e. for each step up in BMI group (e.g. from <25 to 25–29.9 kg/m^2^, or from 25–29.9 to 30–34.9 kg/m^2^) the expected 25(OH)D level decreased by about 1.6 ng/mL. Consequently, a patient in obesity class II (BMI 35–39.9 kg/m^2^) would be expected to have roughly 5 ng/mL lower 25(OH)D than a normal-weight patient, all other variables being equal. Male sex was associated with lower vitamin D levels compared to female sex (B = −2.45; 95% CI, −4.31–-0.58; *p* = 0.010), corresponding to an average difference of approximately 2.5 ng/mL.

Compared with spring, vitamin D levels were significantly higher when blood sampling was performed in autumn (B = 3.84; 95% CI, 1.46–6.22; *p* = 0.002), meaning that measurements taken in autumn were on average almost 4 ng/mL higher than those obtained in spring, after adjustment for other covariates. The differences for summer and winter did not reach statistical significance. Among comorbidities, only COPD was significantly associated with lower vitamin D levels (B = −9.00; 95% CI, −15.94–−2.06; *p* = 0.011), indicating that patients with COPD had 25(OH)D levels almost 9 ng/mL lower than patients without COPD, independent of BMI, sex, season and supplementation.

Age, diabetes, rheumatoid disease, tumour disease, multiple comorbidities, smoking status and the other medication classes showed no independent association with 25(OH)D in the adjusted model (all *p* > 0.05). There was no relevant multicollinearity (all VIF ⩽ 1.5), and residual as well as influence diagnostics (standardised residuals, Cook’s distance) did not indicate major violations of model assumptions.

The detailed parameter estimates are summarised in Table 3.

**Table 3.** Multiple linear regression analysis of determinants of preoperative serum 25(OH)D (ng/mL).

Predictor	B (ng/mL)	95% CI for B	β	*p*-value
Age (per year)	−0.060	−0.148–0.027	−0.049	0.177
BMI category (1–5)	−1.638	−2.524–−0.752	−0.127	**<0.001**
Male sex (ref: female)	−2.447	−4.310–−0.584	−0.092	**0.010**
Summer (ref: spring)	1.540	−1.010–4.091	0.048	0.236
Autumn (ref: spring)	3.840	1.458–6.222	0.129	**0.002**
Winter (ref: spring)	2.022	−0.391–4.434	0.067	0.100
COPD (ref: no comorbidity)	−8.997	−15.938–−2.057	−0.088	**0.011**
Diabetes mellitus (ref: no comorbidity)	−0.982	−3.660–1.696	−0.025	0.472
Rheumatic disease (ref: no comorbidity)	−3.528	−9.803–2.746	−0.037	0.270
Tumour disease (ref: no comorbidity)	−4.628	−10.406–1.149	−0.054	0.116
Multiple comorbidities (ref: none)	−2.163	−11.520–7.194	−0.016	0.650
Aromatase inhibitors (ref: no medication)	6.716	−4.510–17.942	0.040	0.241
Glucocorticoids (ref: no medication)	1.649	−2.927–6.225	0.025	0.708
Levothyroxine (ref: no medication)	1.118	−1.532–3.769	0.030	0.408
PPI (ref: no medication)	0.264	−2.361–2.889	0.007	0.844
Mixed medication regimen (ref: none)	2.397	−1.031–5.826	0.050	0.170
Vitamin D supplementation (yes vs. no)	16.140	13.760–18.519	0.472	**<0.001**
Current smoker (vs. non)	0.555	−2.173–3.283	0.014	0.690
Missing smoking information (vs. non)	−3.714	−11.771–4.342	−0.031	0.366

BMI, body mass index; COPD, chronic obstructive pulmonary dissease; PPI, proton pump inhibitor.

Note: values in bold indicate statistical significance.

Model fit: R^2^ = 0.302, adjusted R^2^ = 0.281; F(19, 633) = 14.44, *p* < 0.001; *n* = 653.

## Discussion

In this prospective cohort of 679 patients scheduled for primary total hip arthroplasty, hypovitaminosis D was highly prevalent, affecting 68% of patients living in a central European region with marked seasonal variation in UV exposure. This finding reinforces previous reports describing widespread vitamin D deficiency in orthopedic patient populations and highlights that low vitamin D status remains a substantial comorbidity among patients undergoing elective joint replacement surgery.^13,16,19,24^ Notably, ⅓ of all patients demonstrated deficiency (⩽19 ng/mL), and only 1 in 3 presented with sufficient 25(OH)D levels (⩾30 ng/mL). These results underline the clinical relevance of vitamin D assessment in preoperative workup, particularly in view of prior research linking deficiency to increased hospital stay and postoperative complications following arthroplasty.^1,2,20–22,25,26^

Contrary to expectation, vitamin D levels were not significantly affected by the season of blood sampling, and hypovitaminosis D occurred at similar rates throughout the year. This diverges from earlier studies that reported marked fluctuations in 25(OH)D concentrations, particularly in northern latitudes with limited UVB availability during winter months.^6,11,17^ The absence of seasonal influence in our cohort may reflect an underlying chronic deficiency regardless of weather conditions.^27^ A progressive shift toward indoor lifestyles, reduced outdoor physical activity in patients with advanced osteoarthritis, and increasing daily screen exposure may contribute to insufficient cutaneous vitamin D synthesis even during summer months. Moreover, older patients with multiple comorbidities may already receive year-round supplementation, further blunting potential seasonal differences. These results suggest that sunlight exposure alone is unlikely to compensate for inadequate levels in this population, supporting the role for routine screening and supplementation independent of season.

We observed that men had significantly lower 25(OH)D levels and a higher prevalence of hypovitaminosis D compared with women. Similar trends have been reported previously in orthopedic and general populations, though mechanisms remain multifactorial.^6,17^ one possible explanation is that women, partly due to long-standing awareness of postmenopausal bone health and osteoporosis risk, may be more frequently screened in primary care settings and consequently more likely to receive supplementation prior to surgery.^28^ Lifestyle differences may further contribute. Women may spend more time outdoors recreationally, engage more regularly in preventive healthcare, or follow dietary supplementation routines more consistently, whereas men in this age group may exhibit lower health-seeking behaviour. Importantly, male sex remained an independent predictor of hypovitaminosis D in our multivariable models, emphasising that male sex represents an independent risk factor for vitamin D deficiency in arthroplasty patients.

In line with prior literature, rising BMI was strongly associated with lower 25(OH)D serum concentrations.^29,30^ With each incremental BMI category, vitamin D levels declined by approximately 1.6 ng/mL, and the odds of sufficiency decreased by 26%. Mechanistically, vitamin D is lipophilic and sequestered in adipose tissue, resulting in reduced bioavailability in overweight and obese individuals.^31^ Additionally, obesity is frequently linked with sedentary lifestyle and decreased outdoor exposure, further impairing vitamin D synthesis. Our findings therefore identify obesity, particularly in combination with male sex, as a readily identifiable high-risk phenotype, warranting targeted supplementation strategies in the context of preoperative optimisation.

Preoperative vitamin D supplementation demonstrated a striking effect. Supplemented patients had mean levels 16 ng/mL higher, and sufficiency was over 5 times more likely than in non-supplemented individuals. This highlights supplementation as both effective and clinically relevant in routine care, in agreement with previous reports from arthroplasty cohorts.^29^,^30^ Interestingly, this subgroup displayed a slightly increased length of stay, which is most likely attributable to the substantially higher mean age rather than vitamin D therapy itself. As the study was observational, residual confounding cannot be excluded and causality cannot be inferred; however, the magnitude of the association strongly supports systematic preoperative correction of deficiency. The finding confirms routine supplementation as a simple, inexpensive intervention capable of correcting deficiency rapidly before surgery.

Surprisingly, all 4 patients with advanced chronic kidney disease (eGFR < 29 mL/min/1.73 m^2^) presented with sufficient vitamin D levels. Although numbers are small, this deviation from expected physiology may reflect closer medical monitoring and more frequent prescription of vitamin D analogues in this high-risk patient group. Indeed, 3 of these patients already reported vitamin D supplementation. Other comorbidities, including diabetes, rheumatic disease and tumour history, were not independently associated with vitamin D status, suggesting that systemic metabolic disease alone does not fully determine deficiency within this population. This is consistent with our multivariable analyses, where comorbidity and general medication classes did not emerge as independent predictors once BMI, sex and supplementation were taken into account.

Baseline WOMAC and EuroQol scores were moderately impaired, consistent with end-stage osteoarthritis, but did not correlate with vitamin D levels. This contrasts with earlier studies reporting associations between deficiency and reduced physical performance or worse preoperative status.^20–22,32^ The lack of correlation in our cohort may suggest that pain and functional limitations at the time of arthroplasty may be predominantly driven by mechanical joint degeneration rather than micronutrient status. However, WOMAC and EuroQol data were only available in ⅔ of patients, which may have limited statistical power to detect small effect sizes.

This study highlights the need for systematic preoperative vitamin D assessment, particularly in obese and male patients. Given the high prevalence of deficiency and the strong effect of supplementation, routine perioperative vitamin D optimisation could represent a meaningful component of prehabilitation. Integrating a simple screening and substitution algorithm into standard arthroplasty pathways may help to address this modifiable risk factor with minimal logistic effort. Whether correction of deficiency translates to improved postoperative outcomes, including earlier mobilisation, reduced infection rates or implant survival, warrants further investigation.

Several limitations must be acknowledged. The study was conducted at a single centre and may not fully represent broader geographic or demographic variations. Postoperative clinical outcomes were not evaluated beyond length of stay, which limits conclusions regarding the impact of vitamin D on rehabilitation, implant longevity or complication rates. Furthermore, seasonal UV exposure and lifestyle behaviors were not assessed, limiting interpretation of seasonal effects. Vitamin D status was analysed at a single preoperative time point, without information on long-term supplementation adherence or changes over time.

Longitudinal studies with postoperative follow-up should assess whether preoperative correction of vitamin D deficiency influences pain, mobility, complication risk or revision-free survival. Future work may also explore personalised supplementation strategies, particularly for obese male patients and those with limited sunlight exposure. Additionally, integrating vitamin D screening into routine arthroplasty pathways may be a feasible step toward reducing modifiable risk prior to surgery. Randomised controlled trials comparing standardised supplementation protocols with usual care are needed to clarify whether optimising vitamin D status translates into clinically meaningful improvements in arthroplasty outcomes.
